# The Serological Test for the Blood

**Published:** 1911-05-06

**Authors:** 


### PATHOLOGY.

### THE SEROLOGICAL TEST FOR THE BLOOD.

The new serological or precipitin test for the detection of the human or other source of mammalian bloodstains has reached the stage of being directly applicable in practice. A practical demonstration of the technique was given recently at the Serological Laboratories of the Royal Institute of Public Health.

In testing the nature of a bloodstain thought to be human a small quantity of the dried bloodstain was scraped from the cloth upon which it had been found and transferred to a test tube containing normal saline solution. Into a second tube was put a more dilute solution than that in the first. Into other tubes were put small quantities of blood derived from a horse, pig, and an ox respectively as controls. Some anti-human serum—that is to say, serum derived from a rabbit into which human blood had been injected under the special conditions —was then put into each of the tubes, with the result that the first and second gave the specific reaction, whilst those containing blood derived from animals other than man, gave no reaction at all. The test consists in the formation of a white cloudy ring which appears almost at once in the strong solution, but only after a minute or two in the weaker. The great point about the test is that it is specific. If a rabbit has been injected with the blood of an ox, its serum may be spoken of as "anti-ox serum," and when mixed with a solution derived from ox blood it gives a white precipitate with this, but gives none with human blood, horse blood, pig blood, and so on; similarly if a rabbit has been injected with dog blood under special circumstances the serum of that rabbit may be described as "anti-dog serum," and if it is put into a series of tubes containing solutions of blood derived from various animal sources it will give the precipitin reaction only in that tube which contains blood derived from a dog, and so on. Sensitising the rabbits is rather a laborious process, but it is possible to obtain a series of rabbits of which the first may for instance supply anti-human, the second anti-horse, the third anti-ox serum, the fourth anti-pig serum and so on, so that in a case of doubt as to the exact source of a given bloodstain it may in this way be possible to determine not merely whether it is mammalian or whether it is derived from human or an animal source, but even the exact animal from which it has come. The far-reaching importance of this in cases of supposed murder is very obvious.

